# Who’s holding the baby? A prospective diary study of the contact patterns of mothers with an infant

**DOI:** 10.1186/s12879-017-2735-8

**Published:** 2017-09-20

**Authors:** Patricia Therese Campbell, Jodie McVernon, Niraj Shrestha, Paula M. Nathan, Nicholas Geard

**Affiliations:** 1 Peter Doherty Institute for Infection and Immunity, University of Melbourne, Parkville, VIC Australia; 2 Infection and Immunity, Murdoch Childrens Research Institute, Royal Children’s Hospital, Parkville, VIC Australia; 3 Melbourne School of Population and Global Health, University of Melbourne, Parkville, VIC Australia; 4 School of Computing and Information Systems, Melbourne School of Engineering, University of Melbourne, Parkville, VIC Australia

**Keywords:** Social networks, Socioeconomic factors, Population characteristics, Infants, Contact patterns

## Abstract

**Background:**

Models of infectious disease are increasingly utilising empirical contact data to quantify the number of potentially infectious contacts between age groups. While a growing body of data is being collected on contact patterns across many populations, less attention has been paid to the social contacts of young infants. We collected information on the social contacts of primary carers of young infants and investigated their potential for use as a proxy for contacts made by their infant.

**Methods:**

We recruited primary carers of infants under one year of age residing in two geographically, demographically and socioeconomically distinct local government areas of Melbourne, Australia — Boroondara and Hume — including a sub-group of Turkish-speaking participants. Participants recorded their own contacts in a paper diary and noted whether their infant was present or absent. Information collected included times at an address; description of location; and details on people contacted at the location. Descriptive summary measures and distributions of contacts by location type, intensity, day of contact and by age are reported.

**Results:**

Of the 226 participants recruited, 220 completed diaries were returned. Participant contact patterns were similar across all groups, with respect to the types of locations, intensity and day of contact, with some variation in the number of unique daily contacts. The infant was present at around 85% of locations at which the primary carer contacted other individuals. The majority of contacts occurring when the infant was present were in Own Home (32%), Retail and Hospitality (18%) and Transport (18%) settings. The mean daily number of unique contacts by infants was estimated as 9.1, 8.7 and 6.5 in Boroondara, Hume (English) and Hume (Turkish), respectively, with a similar age distribution across each of our surveyed groups.

**Conclusions:**

Our demonstration that contact patterns of mothers with infants are reasonably robust to socioeconomic and cultural differences is a step forward in modelling infectious disease transmission. With infants spending most of their time in the company of their mother, contact patterns of mothers are a useful proxy measure of infant contact patterns. The age distribution of contacts made by infants estimated in this study may be used to supplement population-wide contact information commonly used in infectious disease transmission models.

**Electronic supplementary material:**

The online version of this article (10.1186/s12879-017-2735-8) contains supplementary material, which is available to authorized users.

## Background

Infectious diseases are a leading cause of morbidity and mortality in young infants [1]. This high disease burden reflects both immunological naivety, and limited functional reserve, resulting in a greater likelihood of severe disease outcomes, given infection [2]. Public health preventive efforts focus on distinct but complementary approaches: (i) direct protection, achieved by passive (maternal) or active (neonatal and infant) immunisation and (ii) indirect protection or ‘herd immunity’, achieved by vaccine strategies that reduce pathogen circulation and subsequent exposure risk [3].

The potential impact of infectious disease control efforts is often assessed using mathematical models to predict the likely reduction in disease incidence achievable under alternative vaccine strategies. Contact matrices, that quantify the number of potentially infectious contacts between different age groups in a population, are pivotal to model-based estimation of indirect protection. A robust quantitative understanding of contact patterns is therefore essential to ensure the accuracy of these estimates [4].

Studies from the United States indicate that cultural group and family size are strong determinants of infant pertussis infection [5]. However, while a growing body of data is being collected on contact patterns across many populations (e.g. [6–8]), only limited attention has been paid to the social contacts of young infants (e.g. [9, 10]). We have previously reported marked differences in small area-level contact patterns in Greater Melbourne, Australia, associated with socio-economic and cultural diversity [11]. No studies to date have specifically addressed place-based variability in infant contact profiles that might mediate disparate health outcomes.

In this study, we seek to better characterise the contact patterns of primary carers of infants and investigate their use as a proxy measure of infant contacts. We recruited participants from two local government areas (LGAs) of metropolitan Melbourne that differ markedly in geographic, demographic and socioeconomic characteristics. In addition, a nested sub-study focused on households of Turkish origin (the majority culturally and linguistically diverse population) within one of the study areas. The resulting characterisation of contact patterns relevant to very young infants will allow more accurate parameterisation of infectious disease models, particularly those seeking to assess heterogeneity of transmission risk and potential inequities in intervention impact among this critical age group.

## Methods

### Study population

Study participants were recruited from two Melbourne LGAs, Boroondara and Hume. Eligibility criteria included residence in the target LGA, being the primary carer of an infant aged less than one year old at the time of recruitment, and provision of written informed consent to study participation. Participants in Boroondara were recruited between October 2011 and February 2012. Participants in Hume were recruited between September 2013 and July 2014. While the population sizes of these areas are similar, they are otherwise geographically, demographically, and socioeconomically distinct. Hume is located on the fringe of Melbourne, encompassing new growth suburbs and ‘satellite’ townships, 20kms from the central business district (CBD). According to the 2011 census, 31% of its population are aged below 18 years and 43% were born overseas (predominantly Turkey and Iraq) [11]. Median household income for families with children was approximately $1300 per week. Boroondara is located 5 km from the CBD and contains a number of more established suburbs. Compared with Hume, its population is older (21% aged less than 18 years) and only 34% of residents are born overseas (predominantly China and the United Kingdom) [11]. Median household income for families with children in 2011 was nearly double that of Hume, at $2500 per week.

### Survey method

Information on social contacts of study participants was collected using a paper diary modified from European study instruments [6] and evaluated in an earlier pilot study [12]. Participants were recruited through early childhood services and immunisation sessions freely provided by local councils.

Recognising that Hume LGA is home to substantial recent migrant populations, we also sought to recruit study participants from Turkish-speaking households. For this component of the study, all materials were translated into Turkish, and Turkish-speaking research assistants were used to recruit participants.

Participants were randomly allocated two dates on which to complete the study, one week day and one weekend day. A study day commenced on waking and ended with sleep. Our study used a location-based paper diary. Participants were asked to report each change of location made throughout the day on a separate diary page. Each diary page asked a participant to record the arrival time, address, description and departure time, together with the estimated number of people sharing the space with them (with examples provided of 0 if alone in private transport, or 100 if in a crowded movie theatre), and the number at arm’s length (with an example provided of including surrounding seats and aisle on a bus). We defined a *location* as the combination of a physical address, arrival and departure times. We assigned a category of location type (such as Own Home, Work, Study, etc.) to facilitate comparison with similar studies. Participants then recorded a list of their substantive contacts at that location, defined as a two-way or small group conversational exchange involving at least three words or any skin-to-skin contact. Contacted individuals were denoted by first name and initial, and occupation, gender, age and home suburb, where known, were also recorded. The duration and intensity (verbal or physical contact) of each encounter was recorded. Participants were also asked to provide information on the basic demographic characteristics of their household, including the number, age and occupation of family members, and key indicators of household economic status, including income band and housing tenure.

### Data preparation

Collected data were entered into a mySQL database. Addresses of reported locations were checked for accuracy and standardised. Each location was assigned one of ten types (e.g., Own Home, Work, Study, etc.) on the basis of the description provided and allocated a unique identifier. Contacted individuals were also assigned a unique identifier. Time data were checked for logical order and consistency. Further details of data cleaning are provided in Additional file 1.

### Analysis

Participant ages were grouped into five year age blocks from 20 to 44 years. Infant ages were grouped into under two months, two months to less than six months, and six months and over. Contact ages were grouped into five year age groups between 0 and 69 years, and 70 years and over. Participant characteristics were summarised using median (range), mean (standard deviation) or frequency tabulation as appropriate.

The types of locations (e.g. Own Home, Work, Study, etc.) visited by participants were aggregated by population group and stratified by whether or not any contact was reported at the location, and whether or not the infant was present. All contacts made by participants were aggregated by population group and location type, stratified by whether or not physical contact occurred.

The association between demographic and socioeconomic factors and the number of contacts reported on the two collection days (with contacted individuals counted only once per day) was investigated using negative binomial regression.

We defined *companion contacts* as contacts made by the primary carer in the presence of the infant, excluding contacts between the primary carer and the infant. We consider that these companion contacts represent situations in which the primary carer’s reported contacts may best serve as a proxy for contacts made by the infants themselves. For our companion contacts, we report the distribution of visited location types, per person per day. For each population group, we divided the total number of unique reported contacts with each contact age group by twice the number of participants in the population group (to reflect two collection days) to calculate the mean daily number of unique companion contacts with each age group. For our companion contacts, we also report the distribution of times spent with each contacted individual, stratified by whether or not the contacted individual was a household member.

To consider the use of contacts of the primary carer as a proxy measure for the contacts of their infants, we needed to account for the primary carer-infant contact. To do this, we added the age distribution of unique companion contacts to the age distribution of participants, in each population group, to yield a proxy measure of the age distribution of unique infant contacts.

Finally, we report the numbers of unique contacts made each day by participants in Boroondara and Hume (English language group) and compare these to the number of unique contacts made by similarly-aged participants in a telephone survey of the same two LGAs [11].

Analysis was performed using R software, version 3.3.2, The R Foundation for Statistical Computing.

## Results

### Study population

A total of 226 participants (primary carers of an infant aged under 12 months at recruitment) were recruited, 100 from Boroondara and 126 from Hume (101 English language group, 25 Turkish language group). Diaries were returned by 98 participants from Boroondara, 101 participants from Hume (English) and 21 participants from Hume (Turkish). All primary carers were female and identified themselves as the mother of the infant. Participant characteristics are shown in Table 1. Participants from Hume were younger than those from Boroondara, with Hume (Turkish) participants younger than the Hume (English) group. Although a higher percentage of Hume (English) participants owned their home outright or with a mortgage than Boroondara participants, this was not the case for the Hume (Turkish) group. Compared to the Boroondara participants, the Hume (English) group had lower incomes, fewer participants with university qualifications and larger household sizes, with the Hume (Turkish) group more extreme than Hume (English) in these measures.

**Table 1:** Participant characteristics

	Boroondara *n* = 98	Hume – English language *n* = 101	Hume – Turkish language *n* = 21
Sex female %	100	100	100
Median (range) participant age, years	34.7 (25–44)	32 (20–42)	30 (26–42)
Median (range) infant age, days	137 (29–385)	137 (47–327)	177 (14–371)
Household size, mean (SD)	3.6 (0.8)	3.8 (1.0)	4.1 (1.5)
Home owned outright or with mortgage *n* (%)	64 (65%)	87 (86%)	13 (62%)
Renting *n* (%)	32 (33%)	13 (13%)	6 (29%)
Married or living with partner *n* (%)	96 (98%)	96 (95%)	19 (90%)
Income *n* (%) $2000 or more $1600–$1999 $1599 or less	80 (82%) 12 (12%) 6 (6%)	52 (51%) 22 (22%) 27 (27%)	3 (14%) 5 (24%) 13 (62%)
University qualification *n* (%)	77 (76%)	54 (55%)	8 (38%)
Australian born *n* (%)	81 (83%)	88 (87%)	13 (62%)

Due to the delay between the date of recruitment and the data collection dates, three infants from Boroondara and one from Hume (Turkish) were older than one year when data were collected; these infants have been retained in the reported analyses. Infant ages tended to be clustered around vaccine schedule timepoints (2, 4, 6 months) as a consequence of the fact that many participants were recruited at immunisation sessions. Although recruitment for Boroondara occurred over spring/summer, and for Hume over spring/summer/winter, only four participants from Hume were recruited in winter; thus, most participants were reported on contact behaviour at similar times of the year. We therefore anticipate any seasonal differences in weather to have only minimal impact on reported contact behaviour.

### Number and type of locations visited

**Fig. 1:**
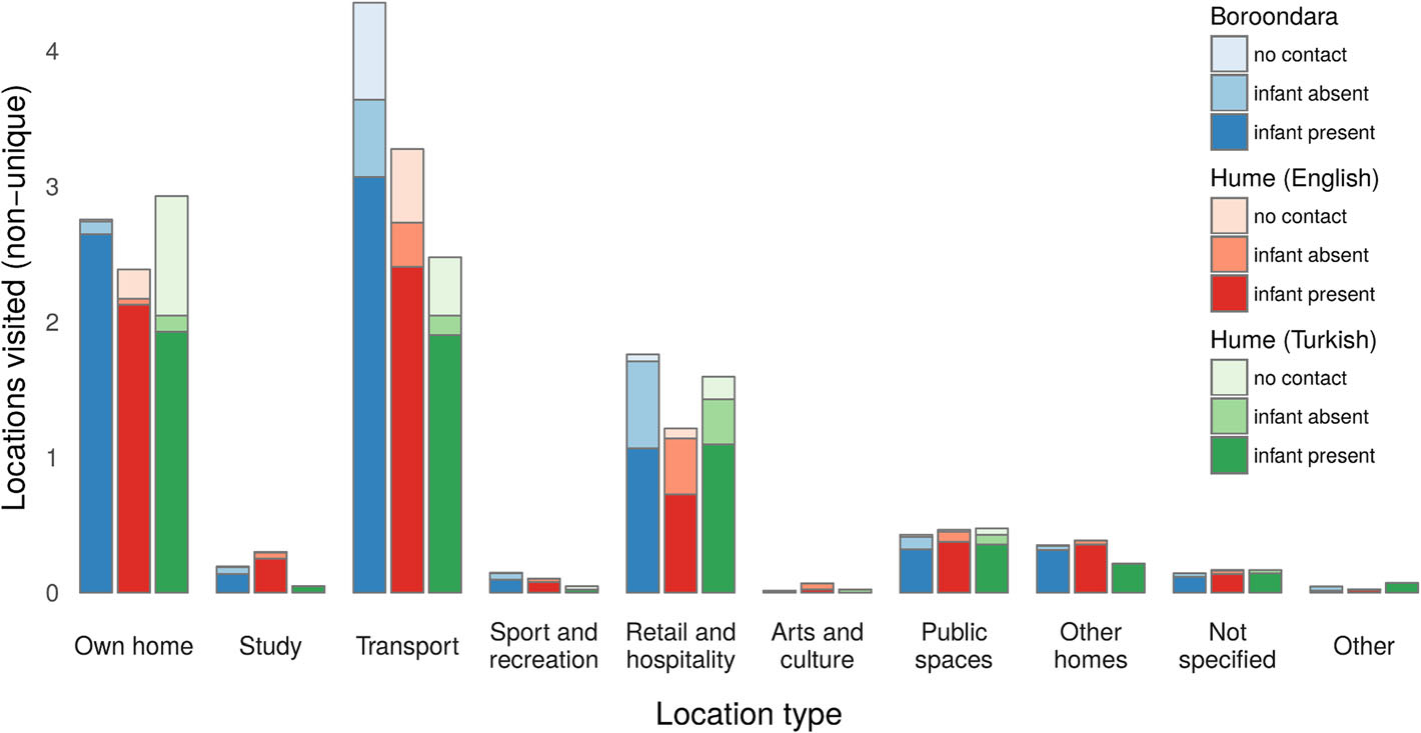
Location types visited by participants. The darkest shade represents locations visited where the infant was present and contact occurred, mid shade locations visited where contact occurred and the infant was absent, and the lightest shade locations visited where no contact occurred

Participants reported visiting 4033 locations (Boroondara, B: 1999; Hume (English), H(E): 1696; Hume (Turkish), H(T): 338), with contact occurring at 3636 of these (B: 1840; H(E): 1523; H(T): 273). For the purpose of understanding patterns of infant contact, we distinguish between locations in which the infant was present, and in which contact was reported to have occurred. Of the locations where contact occurred, the infant was present at 85% or 3088 locations (B: 1530; H(E): 1315; H(T): 243). Note that these locations were not necessarily unique addresses: multiple visits by a participant to the same physical address over the course of the two study days would be recorded as separate locations. Locations were categorised by type, with Fig. 1 showing the distribution of location types for three different contact types: no contact occurred at this location; contact occurred and the infant was present (infant present); and contact occurred and the infant was not present (infant absent). Patterns are similar for each participant group, with Own Home, Transport and Retail & Hospitality (e.g. shops, cafes and restaurants) dominating the visited location types. Boroondara participants visited more Transport location types than either of the two Hume groups.

Similar patterns of location types visited were observed when we restricted the analysis to weekend days only, with the same dominant location types and the infant present for most of the time (Additional file 2). The only exception to this was for the Hume (Turkish) group, who appeared to make more contacts when their infant was absent on weekend days than weekdays.

### Number and intensity of contact events

**Fig. 2:**
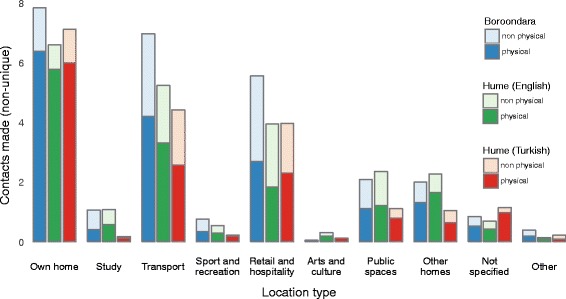
Contacts made by participants, by location type. The daily number of non-unique contacts occurring at each location type, per participant, is shown for each of the three population groups. Contacts are stratified by whether skin-to-skin (physical) or conversational (non-physical) contact occurred

A total of 10,911 contact events were recorded by the 220 participants who completed the study: 5405 by the 98 participants in Boroondara (median (IQR): 52 (39, 69)), 4685 by the 101 participants in Hume (English) (41 (28, 59)) and 821 by the 21 participants in the Hume (Turkish) subgroup (40 (20, 59)). Over 80% of contacts a participant made in their own home involved skin-to-skin contact, while in other settings, such physical contacts accounted for around half to two-thirds of all contacts (Fig. 2).

While living in Boroondara or being English speaking were associated with a higher number of reported contacts (with contacted individuals counted only once per day) in a univariate analysis, only being born in Australia (IRR 1.25 (1.06, 1.48), *p* < 0.01) and having higher income (fortnightly income $2000 or more cf. < $1000 IRR 1.33 (1.04, 1.69), *p* = 0.02) were associated with a higher number of reported contacts in our multivariate negative binomial regression model. Full results are provided in Additional file 3.

To identify companion contacts, we divided contacts into those occurring at locations where the infant was (9522 contacts) and was not (1389 contacts) present with the participant. Of those contact events occurring at locations where the infant was present, 3100 were between the participant and the infant. In total, 6422 contact events (B: 3066; H(E): 2850; H(T): 506) were recorded between the participant and a person other than the infant, in a location where the infant was also present, and thus satisfied our companion contact definition.

### Companion contact patterns

**Fig. 3:**
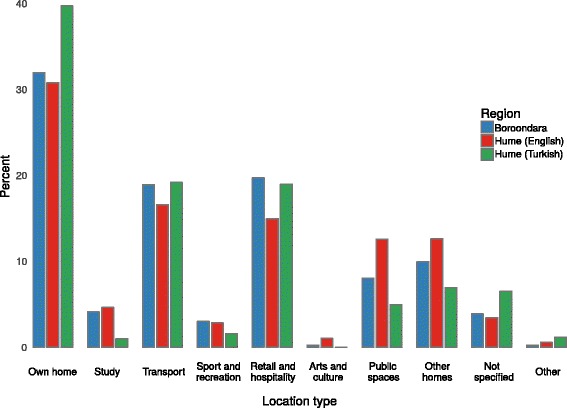
Distribution of companion contact events across different location types. For each of the three populations, the highest percentage of contacts occurred at location types Own Home, Transport and Retail and Hospitality

Following the same trend as observed for all contacts made by the participant, the majority of companion contacts occurred in a participant’s Own Home (32%), Retail and Hospitality (18%) and Transport (18%) location types, with little difference observed between the participant groups, except that Hume (Turkish) participants had a higher percentage of contacts occurring at home (Fig. 3). Unlike contact studies conducted in the general population [6, 11], no contacts were reported in Work location types.

Nearly half of all companion contacts (3080/6422 (48%)) were with members of a participant’s household (B: 1434/3066 (47%); H(E): 1374/2850 (48%); H(T): 272/506(54%)), with statistically significant differences in the proportion of household contacts between the participant groups (*p* < 0.05).

The number of contacts reported on weekdays (3173/6422 (49%)) was similar to that for weekend days (3249/6422 (51%)), with no difference in the proportion of weekday contacts between the participant groups (*p* = 0.281).

### Number of unique companion contacts

Many participants reported repeated contacts with the same person. Transmission risk for readily contagious diseases such as respiratory infections is anticipated to scale with the number of unique sources of potential infection. When excluding repeated contacts, we observed 3310 unique companion contacts (B: 1571; H(E): 1525; H(T): 214). The mean daily number of companion contacts for participants from Boroondara was 8.1 (SD 4.9) people, Hume (English) 7.7 (SD 6.3) people, and Hume (Turkish) 5.5 (SD 3.1) people. Adding one unique contact per day occurring between the infant and their primary carer to these companion contacts, we estimate the mean daily number of contacts of infants as 9.1, 8.7 and 6.5 in Boroondara, Hume (English) and Hume (Turkish), respectively. The values for Boroondara and Hume (English) are similar to the reported number of contacts for a 0–4 year old in Great Britain in the POLYMOD study (8.9 average contacts), which we expect to be the most comparable setting to Australia.

### Contact patterns of infants, by age

**Fig. 4:**
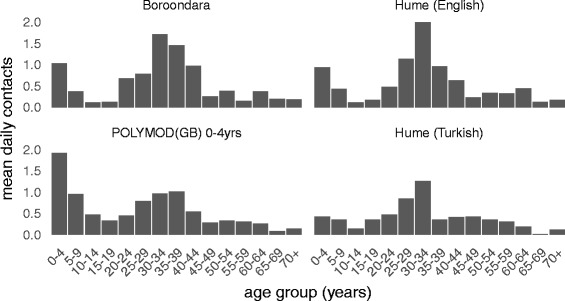
Estimated age distribution of the contacts of infants. The age distribution of infant contacts was derived by supplementing the age distribution of companion contacts with the age distribution of primary carers. The POLYMOD (GB) contact age distribution for the 0–4 year age group, from Table S8.4 in [6] is provided for comparison

We now use companion contacts, supplemented to include primary carer-infant contact, to estimate a proxy measure for the age distribution of contacts made by infants. In all three participant groups, the largest number of these contacts was with people of a similar age to the primary carer (20–39 years) and young children (0–9 years) (Fig. 4). Slight differences observed in the Hume (Turkish) group may reflect the younger ages of the primary carer in this group, which had around 14% of participants aged 35 years or older compared to 50% and 26% in Boroondara and Hume (English), respectively. For reference, Fig. 4 also includes the reported age distribution of contacts made by 0–4 year old children in Great Britain in the POLYMOD study. While the mean daily number of contacts is comparable to that in our study, a major difference is that in the POLYMOD study, children primarily mix with other children, and their parents to a lesser extent. The data containing the mean daily number of contacts made by infants are provided in Additional file 4.

### Time spent with contacts, when the infant is present

**Fig. 5:**
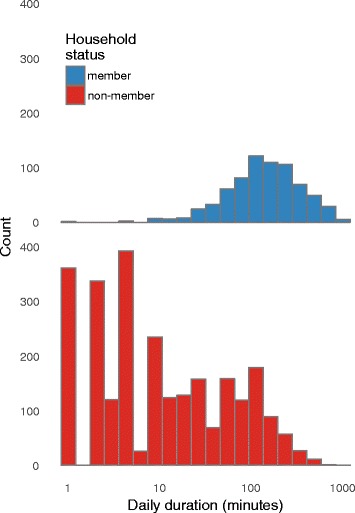
Empirical distribution of the total time spent with each contact during a day. Top panel: household members; Bottom panel: non-household-members. Almost 60% of non-household-contacts involved a total duration of contact of less than 10 min, while total time spent with household members exceeded 10 min for almost all contacts

In Fig. 5, we show the total distribution of time that a participant is in contact with individuals per day, stratified by household member status, when the infant is with them. Empirical distributions of the daily time spent with each contact are provided, showing that while the total amount of time spent with household members tends to be long for most contacts, for almost 60% of non-household members total daily contact is less than 10 min.

### Primary carer contact patterns in the absence of the infant

**Fig. 6:**
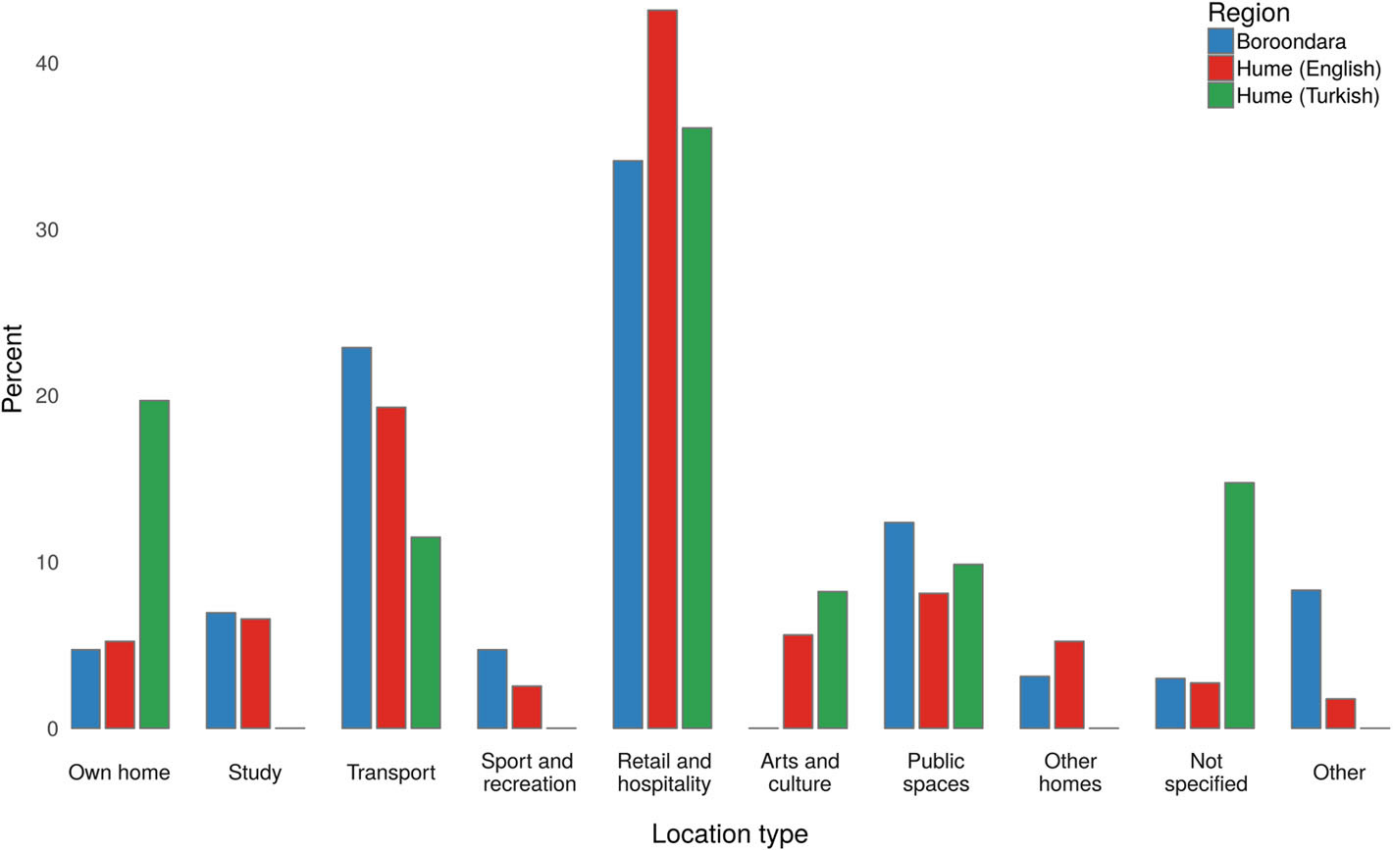
Distribution of non-companion contact events across different location types. The distribution for the Hume (Turkish) group should be interpreted with caution as based on small numbers

Infants were not present in around 15% of locations visited by their primary carers. We have no information about possible contacts made by the infant during these times, however, we do have data on the contact behaviour of their primary carers which represents a potential secondary risk to the infant (*non-companion contacts*). Primary carers made 1389 contacts when their infant was not present, with 972 of these with unique individuals. Note that ‘unique’ here is in reference to non-companion contacts only, and these individuals may have also been contacted when the infant was present. Of the 1389 contact events occurring when the infant was absent, the largest percentages were in Transport, and Retail and Hospitality settings (Fig. 6). In contrast to companion contacts, few contacts were made within the participant’s Own Home when the infant was absent. Values for the Hume (Turkish) group should be interpreted with caution, as only 61 contacts are included.

For both Boroondara and Hume (English), the age distribution of non-companion contacts was similar to that for companion contacts (see Additional file 5 for Supplementary Figure 2, and Additional file 6 for data), with the majority of contacts people of a similar age to the participant, and young children. With 61 contacts, 51 of them unique, numbers were too small in the Hume (Turkish) group to analyse the age distribution of non-companion contacts.

### Primary carer contact patterns compared to an equivalent population without young infants

The study reported here was conducted in conjunction with a broader telephone survey on the contact patterns of residents in Boroondara and Hume LGAs (previously reported in [11]). We compared the contact patterns of primary carers (with or without their infant present) to the contact patterns of the equivalent populations from the telephone survey. Among the 1307 people surveyed in that study, there were 169 women in the age range 20–44 years, 51 in Boroondara and 118 in Hume (English speaking), none of whom lived in households containing an infant aged under one year.

In both Boroondara and Hume, primary carers in the current study reported a greater number of unique contacts per day than the equivalent subset of the population from the telephone survey (Table 2).

**Table 2:** Unique daily contacts of primary carers estimated in this study compared with those of similarly aged females in a telephone survey

	Boroondara median (IQR)	Hume – English language number (IQR)
Infant contact diary study	10 (7–15)	9 (6–13)
Telephone survey	6 (3–11)	5 (3–9)

## Discussion

We collected contact diary data on 220 primary carers of very young infants (under 1 year of age) across three geographically, socioeconomically and culturally diverse populations in Melbourne, Australia. Despite these differences, reported contact behaviour was broadly similar across Boroondara, Hume (English language) and Hume (Turkish language) groups. We found being born in Australia and having a higher income level were associated with having a higher number of contacts, perhaps as these factors may influence the size of a participant’s social network and provide the means for them to participate in social activities.

The design of our study was motivated by the assumption that very young infants spend a large proportion of their time close to their primary carer, and that the contacts of the primary carer might usefully serve as a proxy for infant contacts likely to pose a risk of transmission of respiratory pathogens. The validity of this assumption was borne out by our study: participants reported high levels of contact with their infants, and relatively few locations where the infant was not also present (accounting for around 15% of locations visited).

Young infants, in this study defined as those under one year of age, are an epidemiologically important group due to the elevated risk of severe outcomes from infection. The POLYMOD study groups these very young infants into the 0–4 year age category [6] – a potential limitation for studies that focus on assessing disease risk amongst very young infants. After excluding contacts reported in locations where the infant was not present, we found that the mean number of contacts for 0–1 year olds estimated under our proxy assumption was similar to that observed for the 0–4 year age category in Great Britain in the POLYMOD study [6]. However, the age distribution of contacts by our study and POLYMOD was different, with our infants having contact with relatively fewer young children and more adults than was observed in the POLYMOD study. In settings outside the home, where most of the contacts between participants and other adults occur, an infant is likely to be in very close proximity to their mother due to their own limited mobility. Treating the mother/infant pair as a single entity, our proxy measure of infant contacts therefore reflects the commonly observed phenomenon of assortative mixing between adults. In contrast, the larger 0–4 year age range of the POLYMOD contains children who are more mobile, and even at the same location may spend time away from their mother playing with other children.

We observed that infants are present at locations in which the primary carer makes contact with a considerable number of non-household members, both within the home (i.e. visitors) and outside. While household members do still represent a large proportion of an infant’s potential contacts, the diverse range of other people in proximity to infants raises questions about the likely effectiveness of cocoon vaccination strategies, which aim to prevent infant infection by vaccinating members of their household and close family, assumed to represent the majority of their contacts.

For the primary carers themselves, the common experience of parenthood similarly appears to override any potential effects of socioeconomics and demography. In comparison with similar individuals who did not live in a household containing a young infant (from the telephone survey reported in [11]), the primary carers reported a greater number of contacts. However, it is important to note that the telephone survey collected information retrospectively, which has previously been observed to produce underestimates in contact numbers due to short contacts not being recalled [13, 14].

While the use of companion concepts enables us to comment on the potential contacts of an otherwise difficult-to-survey age group, it also introduces several limitations. In particular, we have limited information on the likely risk of transmission to an infant that is actually posed by a contact reported by their primary carer. The recorded presence of an infant during a primary carer’s contact could correspond to a wide degree of actual risk, ranging from direct contact also occurring with the infant, through to the infant being on the far side of a room when the contact occurred.

Conversely, we have no information on potential contacts made by the infant while they were not with their primary carer, other than those present when the primary carer handed over care of the infant. These contacts will clearly constitute an important element of a realistic population level mixing matrix, and therefore our observed number of contacts made by infants should be treated as an underestimation.

We do not know whether any of the infants were in formal or informal care arrangements during periods of time (15% of reported locations) when they were not with their primary carer, although it would appear that none of the infants utilised formal long day care. Our recruitment process through early childhood services and immunisation clinics, with a requirement that participants were the primary carer of an infant under one year of age, may have missed mothers utilising childcare if another carer brought the infant to clinic, or working mothers were less likely to agree to participate in the study. With 22% of 0–2 year olds in Victoria utilising formal care arrangements [15], our sample may not be representative of all infants, although we expect the use of childcare to be substantially lower for the 0–1 year old age group than for the 1–2 year old age group, reducing the impact of any bias. As patterns of utilisation of out-of-home child care may vary across populations by socioeconomic status [16], this may contribute to differences in exposure risk.

Mathematical models of infectious disease transmission are widely used to estimate the outbreak characteristics and to evaluate the effectiveness of control and mitigation efforts. The usefulness of results derived from these models rests upon the validity of assumptions and data that are used to inform them. Our study provides additional evidence for contact patterns relevant to the transmission of respiratory infection among very young infants. The results of our study suggest that very young infants may experience a considerably higher number of potential contacts compared to previous studies [6, 9]. Our results support previous findings that contact between very young infants and adolescents is limited [9], and that adolescents are therefore unlikely to pose a substantial risk of direct transmission to infants. Nonetheless, the considerable time that very young infants spend outside of their households, and the diverse range of contacts that they are exposed to, highlights the difficulty of insulating these vulnerable infants from potentially infectious exposures. Reducing transmission at the population level, and ensuring early direct protection for infants remain of the utmost importance.

Our data collection method, a location-based prospective paper diary completed by the primary carer over two separate days, was informed by results of a pilot study that found completing a paper diary was acceptable to participants and prospectively collecting information provided more complete data [12]. In a study by Kiti et al. in Kenya, participants too young to record their own contacts were ‘shadowed’ by someone who spent the most time with the participant [10]. Infants reported the fewest contacts in that survey, with the authors acknowledging that some of the contacts occurring while an infant is being held by their mother may have been missed [10]. Further, shadows reported difficulty in keeping track of the participant throughout the day [10]. While our study shares some of these limitations, by collecting contacts of the primary carer, we tried to minimise the shadowing effect.

Our demonstration that the age distribution of contacts in infants less than one year of age is robust to socioeconomic and cultural differences is a step forward in modelling infectious disease transmission involving very young infants. This finding provides confidence that daily numbers of infant contacts with each age group may be used to supplement the population-wide contact information collected in the POLYMOD study [6] to more accurately estimate transmission in infants using mathematical models.

The finding that infants come into contact with more adults than children and with very few adolescents, suggests that targeting vaccination campaigns at adults is likely to be more effective at reducing infant disease incidence than targeting adolescents. Early findings from the United Kingdom’s paediatric influenza immunisation strategy support this view, with immunisation of pre-school aged children resulting in far greater impacts on infant respiratory disease presentations than secondary-school based programs [17]. Focusing such strategies only on household members is likely to have limited success, with around half of infant contacts occurring with non-household members, hence strategies that aim to reduce incidence across the broader population are necessary to effectively protect very young infants.

## Additional files

Additional file 1: Data cleaning. Provides information on the rules used to resolve inconsistencies between duration at a location and duration of recorded contact with a person at the location. (DOCX 14 kb)

Additional file 2: Supplementary Figure 1. Location types visited by participants, for weekend days only. (DOCX 74 kb)

Additional file 3: Supplementary Table 1. Negative binomial regression results. (DOCX 18 kb)

Additional file 4: Mean daily number of contacts of infants. Provides data from our study underlying Fig. 3. (XLSX 10 kb)

Additional file 5: Supplementary Figure 2. Age distribution of unique non-companion contacts. (DOCX 30 kb)

Additional file 6: Mean daily number of non-companion contacts. Provides data from our study underlying Supplementary Figure 1. (XLSX 11 kb)
