# Supplementary material for: Who’s holding the baby? A prospective diary study of the contact patterns of mothers with an infant
Source: BMC Infect Dis. 2017 Sep 20;17:634. doi: 10.1186/s12879-017-2735-8 (PMC5607568; doi:10.1186/s12879-017-2735-8)
Supplement: Supplementary file 1 — Data cleaning. Provides information on the rules used to resolve inconsistencies between duration at a location and duration of recorded contact with a person at the location. (DOCX 14 kb) [file 12879_2017_2735_MOESM1_ESM.docx]

**Who’s holding the baby? A prospective study of the contact patterns of mothers with an infant**

**Additional file 1:** Data cleaning

**1 Time consistency**

Similar to the study by Rolls et al [1], we found a small number of recording inconsistencies between a participant’s time at a location and their time in contact with a person at that location. Clearly the duration of time spent with a contact at a location cannot exceed the duration at that location. As participants recorded in their diary both time of arrival and time of departure at a location, their duration at a location is more reliable than the recorded time spent with a contact at that location. To resolve the inconsistencies between these two measures, we adopted the following rules:

- If a participant’s duration at a location was zero, the allowable duration of contact with a person at this location was capped at 5 minutes (to allow for possible rounding).
- If a participant’s duration at a location was greater than zero, the allowable duration of contact with a person at this location was capped at the duration at the location.

**Reference**

1. Rolls DA, Geard NL, Warr DJ, Nathan PM, Robins GL, Pattison PE, McCaw JM, McVernon J: **Social encounter profiles of greater Melbourne residents, by location – a telephone survey**. *BMC Infectious Diseases* 2015, **15**:494.
