# Supplementary material for: Who’s holding the baby? A prospective diary study of the contact patterns of mothers with an infant
Source: BMC Infect Dis. 2017 Sep 20;17:634. doi: 10.1186/s12879-017-2735-8 (PMC5607568; doi:10.1186/s12879-017-2735-8)
Supplement: Supplementary file 2 — Supplementary Figure 1. Location types visited by participants, for weekend days only. (DOCX 74 kb) [file 12879_2017_2735_MOESM2_ESM.docx]

**Who’s holding the baby? A prospective study of the contact patterns of mothers with an infant**

**Additional file 2:** Location types visited by participants for weekend days only.

**
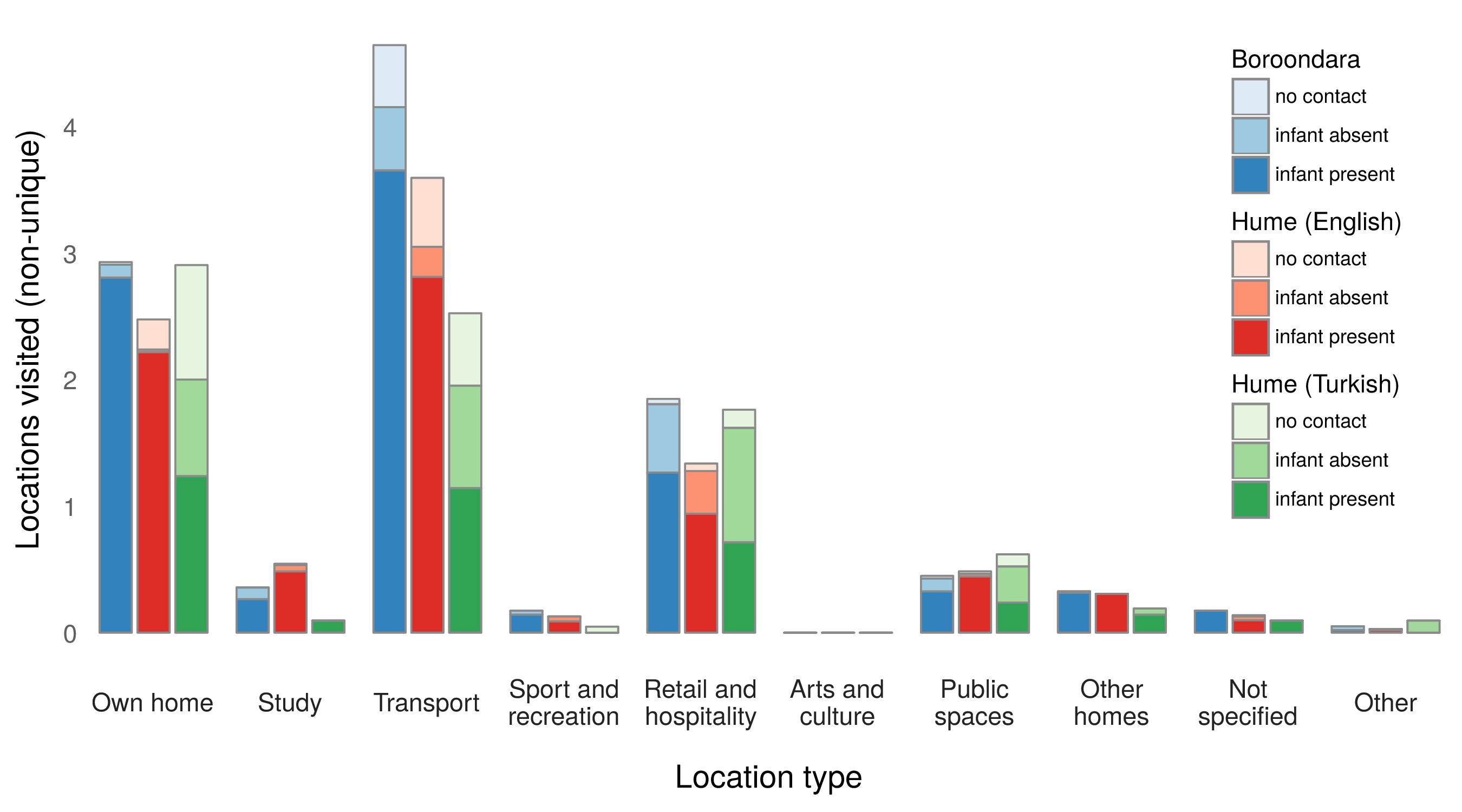
**

**Supplementary figure 1: Location types visited by participants on weekend days.** The darkest shade represents locations visited where the infant was present and contact occurred, mid shade locations visited where contact occurred and the infant was absent, and the lightest shade locations visited when the infant was absent.
