# Supplementary material for: Who’s holding the baby? A prospective diary study of the contact patterns of mothers with an infant
Source: BMC Infect Dis. 2017 Sep 20;17:634. doi: 10.1186/s12879-017-2735-8 (PMC5607568; doi:10.1186/s12879-017-2735-8)
Supplement: Supplementary file 3 — Supplementary Table 1. Negative binomial regression results. (DOCX 18 kb) [file 12879_2017_2735_MOESM3_ESM.docx]

|  | | **Univariate** | | | **Multivariate** | |
| --- | --- | --- | --- | --- | --- | --- |
| **Variable** | | **Number of contacts** | **IRR (95% CI)** | **P value** | **IRR (95% CI)** | **P value** |
| *Household size* | |  |  |  |  |  |
|  | 2 | 15.8 | Ref |  |  |  |
|  | 3 | 19.1 | 1.21 (0.74–1.97) | 0.44 | 1.02 (0.49–2.12) | 0.97 |
|  | 4 | 22.1 | 1.40 (0.86–2.30) | 0.18 | 1.17 (0.56–2.44) | 0.67 |
|  | 5 | 24.1 | 1.53 (0.92–2.53) | 0.10 | 1.35 (0.65–2.83) | 0.42 |
|  | 6–8 | 15.0 | 0.95 (0.53–1.71) | 0.87 | 1.10 (0.47–2.57) | 0.83 |
| *Local Government Area (LGA)* | |  |  |  |  |  |
|  | Boroondara | 22.7 | Ref |  |  |  |
|  | Hume | 19.0 | 0.84 (0.74–0.95) | 0.01 | 0.90 (0.78–1.04) | 0.15 |
| *Mother age (years)* | |  |  |  |  |  |
|  | 20–24 | 15.0 | Ref |  |  |  |
|  | 25–29 | 17.4 | 1.18 (0.58–2.38) | 0.65 | 1.09 (0.54–2.21) | 0.81 |
|  | 30–34 | 21.2 | 1.41 (0.71–2.84) | 0.33 | 1.20 (0.60–2.42) | 0.61 |
|  | 35–39 | 20.9 | 1.39 (0.69–2.81) | 0.35 | 1.09 (0.53–2.23) | 0.81 |
|  | 40–44 | 23.9 | 1.60 (0.78–3.28) | 0.20 | 1.29 (0.62–2.68) | 0.50 |
| *Infant age* | |  |  |  |  |  |
|  | < 2 mths | 20.8 | Ref |  |  |  |
|  | 2 mths – < 6 mths | 20.5 | 0.99 (0.82–1.19) | 0.91 | 0.83 (0.68–1.01) | 0.06 |
|  | 6 mths or older | 20.8 | 1.00 (0.82–1.23) | 0.98 | 0.86 (0.70–1.06) | 0.17 |
| *Country of birth* | |  |  |  |  |  |
|  | Outside Australia | 17.4 | Ref |  |  |  |
|  | Australia | 21.3 | 1.23 (1.04–1.46) | 0.02 | 1.25 (1.06–1.48) | **<0.01** |
| *Language* | |  |  |  |  |  |
|  | English | 21.3 | Ref |  |  |  |
|  | Turkish | 14.2 | 0.66 (0.53–0.83) | < 0.01 | 0.87 (0.68–1.12) | 0.28 |
| *Marital status* | |  |  |  |  |  |
|  | Living with partner | 20.4 | Ref |  |  |  |
|  | Married | 20.8 | 1.02 (0.83–1.25) | 0.88 | 1.05 (0.86–1.29) | 0.60 |
|  | Never married | 26 | 1.27 (0.77–2.10) | 0.35 | 1.23 (0.75–2.02) | 0.41 |
|  | Separated | 13.5 | 0.66 (0.32–1.37) | 0.27 | 1.04 (0.46–2.36) | 0.93 |
|  | Single | 13 | 0.64 (0.35–1.17) | 0.15 | 0.66 (0.29–1.52) | 0.33 |
| *Education* | |  |  |  |  |  |
|  | University | 22.5 | Ref |  |  |  |
|  | TAFE | 17.4 | 0.78 (0.67–0.90) | < 0.01 | 0.81 (0.70–0.94) | <0.01 |
|  | Year 11–Year 12 | 19.7 | 0.88 (0.68–1.13) | 0.31 | 0.93 (0.72–1.18) | 0.54 |
|  | Up to Year 10 | 11.6 | 0.52 (0.33–0.81) | < 0.01 | 0.56 (0.33–0.95) | 0.03 |
|  | Other | 18.0 | 0.80 (0.52–1.22) | 0.30 | 0.71 (0.48–1.06) | 0.10 |
| *Home ownership* | |  |  |  |  |  |
|  | Owned outright | 24.1 | Ref |  |  |  |
|  | Being purchased | 20.5 | 0.85 (0.66–1.10) | 0.21 | 0.80 (0.64–1.01) | 0.07 |
|  | Being rented | 20.4 | 0.84 (0.64–1.11) | 0.23 | 0.83 (0.64–1.07) | 0.15 |
|  | Rent free | 18.5 | 0.77 (0.37–1.57) | 0.47 | 0.62 (0.32–1.20) | 0.16 |
|  | Other | 14.0 | 0.58 (0.28–1.22) | 0.15 | 0.60 (0.29–1.24) | 0.17 |
| *Income (per fortnight)* | |  |  |  |  |  |
|  | < $1,000 | 14.9 | Ref |  |  |  |
|  | $1,000–$1,999 | 19.1 | 1.28 (1.00–1.64) | 0.05 | 1.28 (1.00–1.63) | **0.05** |
|  | $2,000 or more | 22.3 | 1.49 (1.19–1.88) | < 0.01 | 1.33 (1.04–1.69) | **0.02** |

Supplementary Table 1: Outcome of negative binomial regression for the number of contacts recorded by participants over two days, with daily repeated contacts removed.
