# Supplementary material for: Who’s holding the baby? A prospective diary study of the contact patterns of mothers with an infant
Source: BMC Infect Dis. 2017 Sep 20;17:634. doi: 10.1186/s12879-017-2735-8 (PMC5607568; doi:10.1186/s12879-017-2735-8)
Supplement: Supplementary file 5 — Supplementary Figure 2. Age distribution of unique non-companion contacts. (DOCX 30 kb) [file 12879_2017_2735_MOESM5_ESM.docx]

**Who’s holding the baby? A prospective study of the contact patterns of mothers with an infant**

**Additional file 5:** Supplementary figure 2


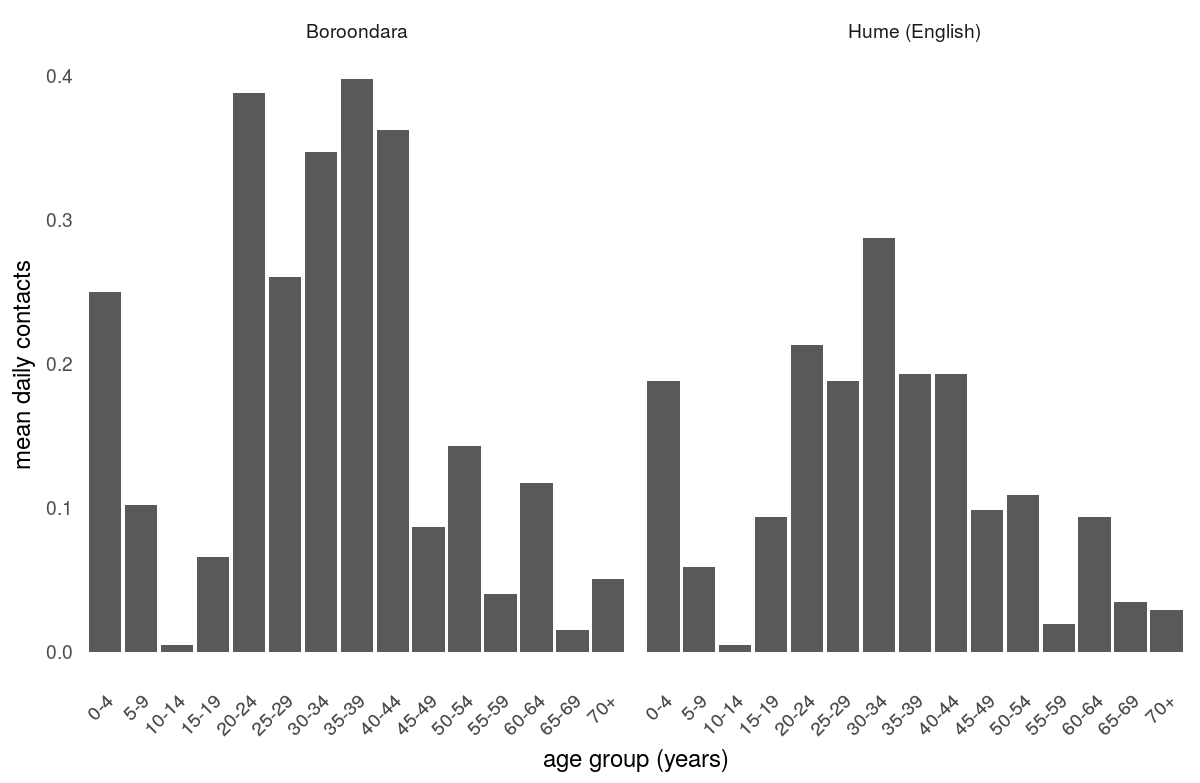


Supplementary Figure 2: Age distribution of unique non-companion contacts for Boroondara and Hume (English). Note the scale difference compared to Fig 4 in the main paper.
